# Disruption or partitioning? Deep eutectic solvent biphasic systems for integrated seaweed biorefineries

**DOI:** 10.1186/s40643-026-01139-9

**Published:** 2026-09-25

**Authors:** Isa S. A. Hiemstra, Wimar Reynaga-Navarro, Rene H. Wijffels, Michel H. M. Eppink, Antoinette Kazbar

**Affiliations:** 1 Bioprocess Engineering, Wageningen University, PO Box 16, 6700 AA Wageningen, The Netherlands; 2 Faculty of Biosciences and Aquaculture, Nord University, 8049 Bodø, Norway; 3 Faculty of Applied Sciences, Department of Biotechnology, TU Delft, Van der Maasweg 9, 2629 HZ Delft, The Netherlands

**Keywords:** Biorefinery, Brown seaweed, Deep eutectic solvents, Alginate, Fucoxanthin

## Abstract

In this study, a DES-based biphasic system (FABU) was evaluated for the multiproduct extraction of compounds from *Saccharina latissima*. The system combined a hydrophobic fatty-acid phase (dodecanoic acid–octanoic acid, FA) for fucoxanthin extraction with a hydrophilic betaine-urea-phase (BU) for alginate recovery, together with citric acid pretreatment for fucoidan and mannitol recovery. While fucoxanthin partitioning into the FA phase was limited (41–58 µg g^− 1^), a substantially higher fucoxanthin concentration (563 ± 8 µg g^− 1^) was recovered during the subsequent alkaline extraction step. This behaviour indicates that the DES-based system likely acts as a medium that enhances biomass permeability rather than directly promoting partitioning. Chromatographic profiles further showed enhanced fucoxanthin preservation relative to conventional pigment extraction, suggesting a protective role of the DES matrix. These findings highlight a decoupling between biomass disruption and solubilisation in DES-based systems. Our research shows new opportunities to deploy biphasic DES systems as powerful pretreatment platforms within integrated seaweed biorefineries, thereby offering a new strategy for preserving and recovering sensitive compounds.

**Graphical abstract:**

**Figure:**
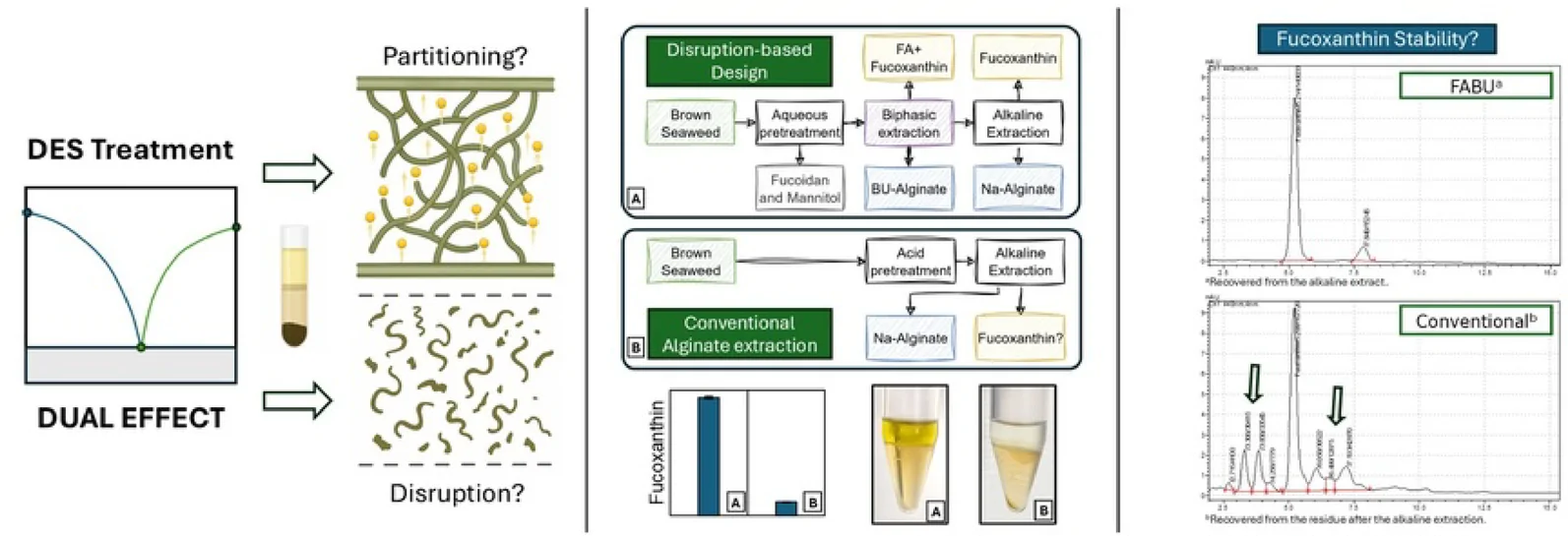

**Supplementary Information:**

The online version contains supplementary material available at https://doi.org/10.1186/s40643-026-01139-9.

## Introduction

Conventional extraction processes often rely on volatile organic solvents and energy-intensive operations, limiting their sustainability (Paiva et al. 2014). Deep eutectic solvents (DES) have emerged as a versatile and potentially sustainable alternative to conventional solvents (Ivanović et al. 2020, Li and Row 2016, Ling and Hadinoto 2022). Formed by the combination of hydrogen bond acceptors (HBA) and hydrogen bond donors (HBD), DES exhibit depressed melting points and tuneable physicochemical properties. Owing to their adjustable polarity, hydrogen-bonding capacity, and compositional flexibility, DES are promising media for selective extraction and biomass fractionation (Hansen et al. 2021, Smith et al. 2014). In recent years, DES have been successfully applied to various biomass types, including plant materials, microalgae, and seaweed, demonstrating enhanced recovery of a wide range of compounds (Zdanowicz et al. 2018, Ozel et al. 2024, Saravana et al. 2018, Hiemstra et al. 2014, Reynaga-Navarro et al. 2026).

Brown seaweeds represent an attractive feedstock for integrated biorefinery concepts due to their diverse chemical composition, including the polysaccharide alginate. Alginate confers unique gelling, viscosity, and chelation properties (Abka-khajouei et al. 2022, Draget 2009). In addition to alginate, brown seaweeds are also rich in other carbohydrates such as fucoidan and mannitol. Fucoidan, a sulphated polysaccharide present together with alginate in the cell wall matrix, possesses reported anti-inflammatory, anticancer, antiviral properties, among others (Li et al. 2008, Wang et al. 2019). On the other hand, mannitol, a sugar alcohol is widely used as a low-calorie sweetener and platform molecule in food and chemical industries (Hosseini et al. 2024). Furthermore, brown seaweed contains the high-value pigment fucoxanthin (Fig. S1). This carotenoid, belonging to the xanthophyll class, possesses reported antioxidant and bioactive properties, making it a promising compound for nutraceutical and functional food applications (Din et al. 2022, Zhang et al. 2015).

Despite the presence of various valuable compounds, current industrial processing strategies remain largely focused on alginate as the primary target product, often relying on harsh alkaline and acid treatments. These conventional approaches involve high chemical consumption and energy input, limiting overall biomass valorisation and underutilising the potential of other valuable components (Draget 2009, Saji et al. 2022). Furthermore, the complex and recalcitrant structure of seaweed biomass presents a significant barrier to efficient disruption and access to intracellular compounds (Saravana et al. 2023, Steinbruch et al. 2023). This limited accessibility further constrains the recovery of valuable components and often requires intensive processing conditions.

As an alternative, DES have been shown to act as effective pretreatment agents, promoting biomass disruption and enhancing the accessibility of target compounds (Loow et al. 2017, Li et al. 2023, Matchim Kamdem et al. 2023). In brown seaweed processing, hydrophilic DES have been shown by our group to enable efficient alginate extraction (Hiemstra et al. 2014) and to modify product functionality, yielding alginates with significantly higher viscosities than those obtained by conventional methods (Reynaga-Navarro et al. 2026). Beyond polysaccharides, hydrophobic DES showed effective extraction of non-polar bioactive compounds, while simultaneously promoting cellular disruption and facilitating polysaccharide release (Hiemstra et al. 2025).

Building on this work, the present study represents the next step in a broader research line on DES-based seaweed biorefineries. It integrates these previously developed technologies into a single, sequential, multiproduct biorefinery process for the simultaneous recovery of multiple seaweed products, thereby potentially intensifying the process.

Our previous findings highlight that DES can go beyond their role as alternative solvents, serving instead as functional media that enable integrated, multiproduct extraction strategies. While single-phase DES systems have primarily been developed for the recovery of individual product streams, the complementary properties of hydrophobic DES for pigment recovery and potential membrane disintegration, and hydrophilic DES for cell wall disruption and the selective extraction of high-viscous alginate, suggest the feasibility of a unified, biphasic approach. The integration of these two phases allows alginate and fucoxanthin to be selectively partitioned within the same process. Furthermore, a preceding aqueous pretreatment enables the recovery of the readily water-soluble fucoidan and mannitol. Such a biphasic system exemplifies process integration and intensification by combining extraction and selective partitioning within a single workflow, thereby reducing the need for separate extraction processes and advancing toward full biomass valorisation in an integrated seaweed biorefinery.

Although promising, biphasic DES systems for multiproduct seaweed fractionation remain largely unexplored. Critical aspects such as phase interactions, extraction parameters, and component partitioning behaviour are not yet investigated. We hypothesise that the complementary properties of hydrophobic and hydrophilic DES enable selective partitioning of compounds with contrasting polarities, thereby integrating pigment recovery and polysaccharide extraction within a single process.

## Materials & methods

### Biomass

*Saccharina latissima* was harvested in May 2024 from the Brittany region (France) and delivered frozen (− 20 °C). The biomass was subsequently oven-dried for 72 h in an industrial oven (Marius Engineering, Netherlands) without light exposure. The dried material was milled using a Retsch^®^ SM300 grinder fitted with a 1 mm sieve and stored at 4 °C until further use.

### Biomass characterisation

Alginate, fucose, mannitol, and fucoxanthin contents were determined in the dried *S. latissima biomass*, with a dry weight content of 93.6%. Alginate was quantified by methanolysis of the dried biomass (Reynaga-Navarro et al. 2026), followed by analysis using ion-exchange chromatography (Sect. 2.3.2). Fucose and mannitol were determined after trifluoroacetic acid (TFA) hydrolysis of 2 mg of dried biomass and subsequent ion-exchange chromatography analysis. Fucoxanthin was quantified by extraction with methanol containing 10% water v/v at 25 °C for 2 h using a SLR of 0.05 g mL^− 1^ (Kholany et al. 2025).

Alginate, fucose, and mannitol contents were 296 ± 10, 10 ± 1, and 73 ± 3 mg g^− 1^ DW, respectively, while the conventional methanolic extraction recovered 338 ± 65 µg g^− 1^ DW of fucoxanthin.

### DES preparation

DES were prepared by mixing the compounds (HBA and HBD) in their corresponding molar ratio in a water bath at 60 °C until a clear liquid was formed (Table 1). Water was added to the hydrophilic DES to reach the desired water content.

**Table 1:** DES composition and characteristics

DES nature	HBA	HBA	Ratio	Water%	Abbreviation
Hydrophobic	Dodecanoic acid	Octanoic acid	1:1	–	FA
Hydrophilic	Betaine	Urea	1:2	25, 45 and 65%	BU

### Analytical methods and yields

#### Alginate quantification

Alginate extracts (1 mL) were mixed with 2 mL of ethanol-water mixture 80% v/v with 1% NaCl w/v for alginate precipitation. The samples were centrifuged at 4255 *g* for 10 min at RT (from now on these conditions will remain unless stated otherwise), the supernatant discarded and the pellet washed with 1 mL of ethanol-water mixture 80% v/v. The samples were centrifuged, the supernatant discarded and the pellets were dried with a gentle stream of nitrogen. The dried crude alginate pellet was dissolved in milli-Q water for analysis.

The alginate concentration was determined via the colorimetric method described by (Cesaretti et al. 2003). The extraction yield *Y*_*alg*_ (mg g-1) express as dry basis is determined as follows:

1 $$ Y_{{alg}} = \frac{{C_{{UA}} \cdot V \cdot 0.907}}{{W_{{sw}} \cdot x_{{dw}} }} $$

where *C*_*UA*_ is the concentration of uronic acids (mg mL^− 1^), *V* is the volume of the sample (mL), *W*_*sw*_ is the amount of seaweed (g) and *x*_*DW*_, and 0.907 is the correction factor to convert the concentration of uronic acids into alginate. D-galacturonic acid (≥ 97% purity, Sigma-Aldrich) was used as a standard.

#### Ion exchange chromatography for fucose and mannitol

For fucoidan analysis, an aliquot of 1 mL of extract was mixed with 2 mL of pure ethanol and let overnight at 4 °C. Then, the mixture was centrifuged, the supernatant discarded and the pellet washed with 1 mL of ethanol 80% v/v. Finally, the pellet was dried with a stream of nitrogen. The dried pellet was then hydrolysed with 1 mL of Trifluoracetic acid (TFA) 2 M for 90 min at 120 °C, cooled down and later the TFA was evaporated with a nitrogen stream. The dried sample is then dissolved in milli-Q water, filtered and 10 µL injected into a Dionex ICS-6000 HPIC System (Thermo Fisher Scientific™) at 30 °C in a flow rate of 0.5 mL min^− 1^ through a Dionex Carbopac™ PA20 (3 × 150 mm) column from ThermoFisher Scientific™ using 100 mM NaOH (A), 500 mM NaOH (B), 500 mM sodium acetate + 50 mM NaOH (C) and Milli-Q water (D) as eluents, following a protocol described previously (Reynaga-Navarro et al. 2026). Fucose content was used as a proxy of the fucoidan content. For mannitol determination, the extract was filtered and 10 µL were injected into ion exchange chromatographer as described previously in this section. Fucose (≥ 99%, Supelco) and mannitol (≥ 99.9%, Supelco) were used as standards. Yields (*Y*_*s*_) were expressed as follows:

2 $$ Y_{s} = \frac{{C_{s} \cdot V}}{{W_{{sw}} \cdot x_{{dw}} }} $$

where *C*_*s*_ is the monomer concentration (either fucose or mannitol), *V* is the volume of the sample, *W*_*sw*_ is the weight used for the extraction and *x*_*dw*_ is the fraction of dry weight.

#### Fucoxanthin determination

The fucoxanthin concentration was determined by ultra-high-performance liquid chromatography with diode array detection (UHPLC-DAD) using a Shimadzu Nexera X2 system (Kyoto, Japan). Samples (20 µL) were injected into a Kinetex^®^ C18 column (5 μm, 100 Å, 150 × 4.6 mm; Phenomenex^®^) at a flow rate of 1 mL min^− 1^. The mobile phases consisted of (A) 0.5 M ammonium acetate in methanol: Milli-Q water (85:15, v/v), (B) 90% aqueous acetonitrile, and (C) 100% ethyl acetate. The total run time was 53 min, following the gradient programme described by (Kholany et al. 2025). Acetonitrile, ethyl acetate, and methanol (Lichrosolv^®^, analytical grade) and ammonium acetate (ultra-pure) used for mobile phase preparation were purchased from Supelco™ and VWR Chemicals™, respectively. All-trans fucoxanthin (≥ 95% purity, Supelco™) was used as the external standard. The fucoxanthin extraction yield Y_F*x*_ (µg g^− 1^ dry weight) was calculated as follows:

3 $$ Y_{{Fx}} = \frac{{C_{{Fx}} \cdot V}}{{W_{{sw}} \cdot x_{{dw}} }} $$

where *C*_*Fx*_, is the concentration of fucoxanthin in the sample, *V* is the volume of the sample *W*_*sw*_ is the weight used in the extraction and *x*_*dw*_ is the dry weight of the sample.

### Acid-alkaline extraction as control

The seaweed biomass was mixed with a 0.2 M H_2_SO_4_ solution at a SLR of 1:60 (g mL^−1^) and pretreated for 1 h at room temperature under constant mixing. The mixture was subsequently neutralised using a 3.25% (w/w) sodium carbonate solution until reaching pH 10–11 and then incubated at 55 °C for 1 h to promote alginate extraction. Later, the mixture was cooled to room temperature and centrifuged, and the supernatant was collected for alginate analysis.

### Preliminary test of biphasic system

To evaluate the technical feasibility of the biphasic system forming, mixing tests of FA DES with BU DES (from now on FABU) in weight ratios of 1:2, with different concentrations of water (25, 45, 65%). Mixtures were centrifuged and the biphasic system formation was evaluated visually.

### Biphasic extraction process

Later, alginate extractions were performed. Dry seaweed was mixed with FABU in a SLR of 1:60 w/w. Extraction was performed at 35 °C for 1 h with constant agitation. Then, the mix was centrifuged (4255 *g* for 10 min). Subsequently, the phases were separated, and the BU phase was collected for alginate quantification.

This integrated biphasic extraction system was directly adapted from the platforms previously developed and validated. The hydrophilic phase configurations for high-viscosity alginate extraction and the hydrophobic phase configurations for pigment recovery are summarized in Table 2.

**Table 2:** Biphasic system DES phases based used

DES phase	Components	Target compound	References
Hydrophilic	Betaine: urea (1:2)	High-viscosity alginate	Reynaga-Navarro et al. (2026)
Hydrophobic	Dodecanoic acid: octanoic acid (1:1)	Fucoxanthin	Hiemstra et al. (2025)

### Pretreatments evaluation in combination with the biphasic system

**Fig. 1:**
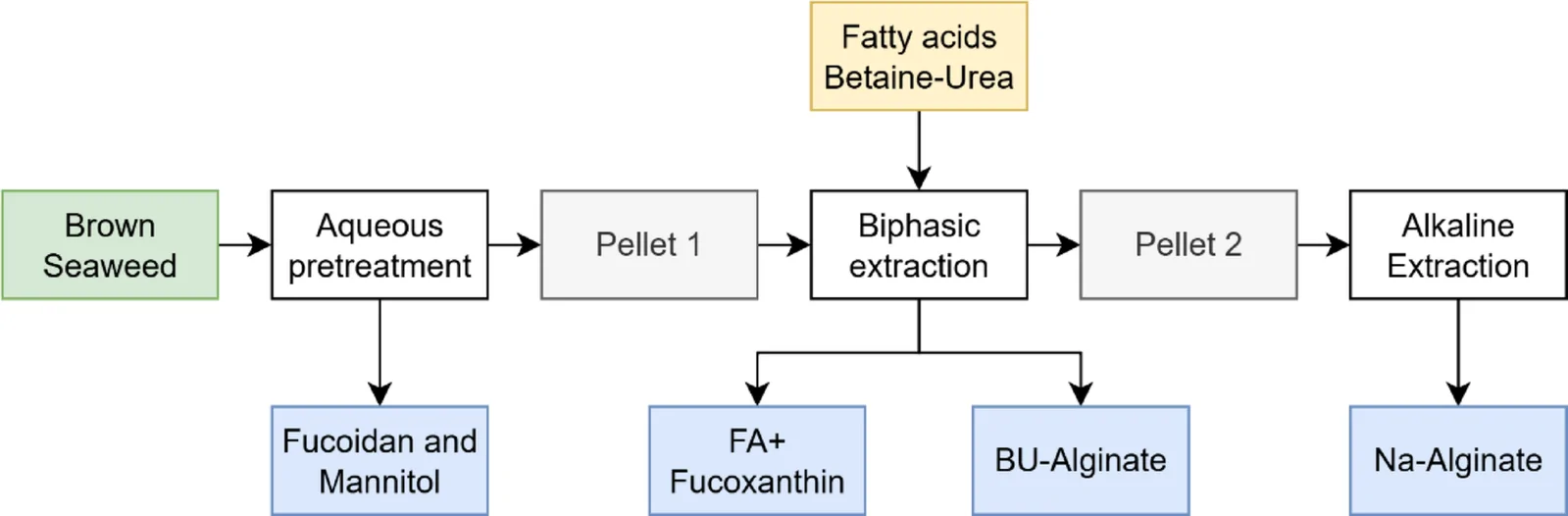
Flowchart of the experimental design combining the pretreatment and the biphasic system

Pretreatments were evaluated in combination with the biphasic extraction process (Fig. 1). These pretreatments also enabled the recovery of water-soluble compounds. Two pretreatment conditions were tested: (i) extraction with water at 35 °C for 1 h and (ii) extraction with a 50 mM citric acid solution at 35 °C for 1 h (Fawzy and Gomaa 2021). After each pretreatment, the mixture was centrifuged and the supernatant collected for the analysis of fucose and mannitol. The resulting wet pellet was then mixed with the biphasic extraction system while maintaining a SLR of 1:60, and the extraction was performed at 35 °C for 1 h. Following centrifugation, the fatty acid phase and the betaine-urea phase were collected and analysed for fucoxanthin and alginate, respectively.

The remaining pellet was subsequently mixed with water and sodium carbonate to reach a final Na_2_CO_3_ concentration of 0.1 M (pH 10–11), adjusting the volume to maintain the original SLR. Alkaline extraction was then performed at 55 °C for 1 h. The mixture was centrifuged and the supernatant collected for alginate analysis. All extractions were performed under light protected conditions.

### Fucoxanthin recovery from the alkaline extract after the biphasic system

The alginate extract obtained after the alkaline step from the biphasic system pellet was subjected to liquid–liquid extraction with ethyl acetate at a volumetric ratio of 1:1. The mixture was vortexed and subsequently centrifuged at 21,000 *g* for 5 min at 4 °C. After phase separation, the upper organic phase was collected, diluted with methanol, and analysed by chromatography (see Sect.  2.3.3).

### COSMO-RS analysis

The COSMO-RS (Conductor-like Screening Model for Real Solvents) approach was employed to predict the thermodynamic behaviour of the studied DES systems for fucoxanthin partitioning (Xu et al. 2022) All calculations were carried out using COSMO-RS version 2022.103 (Software for Chemistry and Materials BV, Amsterdam, The Netherlands), implemented within the Amsterdam Density Functional (ADF) framework. Prior to COSMO-RS analysis, molecular structures were geometry-optimised using the universal force field (UFF), followed by density functional theory (DFT) calculations at the GGA: BP86 level with a TZP basis set (Hiemstra et al. 2026).

Using COSMO-RS, the activity coefficient of alginate at infinite dilution was calculated according to Eqs. 4 and 5:

4 $$ {\mathrm{ln}}\left( {\gamma _{i}^{\infty } } \right) = \frac{{\mu _{s}^{i} - \mu _{i}^{i} }}{{RT}} $$

5 $$ \gamma _{i}^{\infty } = limit_{{xi \to 0\gamma _{i} }} $$

where $\gamma _{i}^{\infty }$ represents the activity coefficient of fucoxanthin (*i*) at infinite dilution, defined as the limiting value of the activity coefficient as the solute concentration approaches zero (Hiemstra et al. 2026). It is determined based on the chemical potential of the solvent $\mu _{s}$ and the chemical potential of the pure compound $\mu _{i}$ (Ozturk and Gonzalez-Miquel 2019, Al-Maari et al. 2024).

With the activity coefficient, the solubility capacity $\left( {C_{i}^{\infty } } \right)$ of the DES was calculated to determine the maximum amount of solute that can be dissolved in the DES, following Eq. 6 (Xu et al. 2022):

6 $$ C_{i}^{\infty } = 1/\gamma _{i}^{\infty } $$

The partition coefficient was calculated to assess the distribution of the solute between the two phases (Hiemstra et al. 2026), providing insights into the partitioning behaviour during the biphasic extraction. The partition coefficient (${\mathrm{K}}$) was calculated using Eq. 7:

7 $$ K_{{FA/BU}} = \frac{{\frac{1}{{\gamma _{i}^{\infty } \left( {FA} \right)}}}}{{\frac{1}{{\gamma _{i}^{\infty } \left( {BU} \right)}}}} $$

### Statistical analysis

One-way ANOVA was performed to compare the experimental groups. When significant differences were detected (*p* < 0.05), Tukey’s post hoc test was applied to determine pairwise differences between groups.

## Results & discussion

### Aqueous seaweed pretreatment

**Fig. 2:**
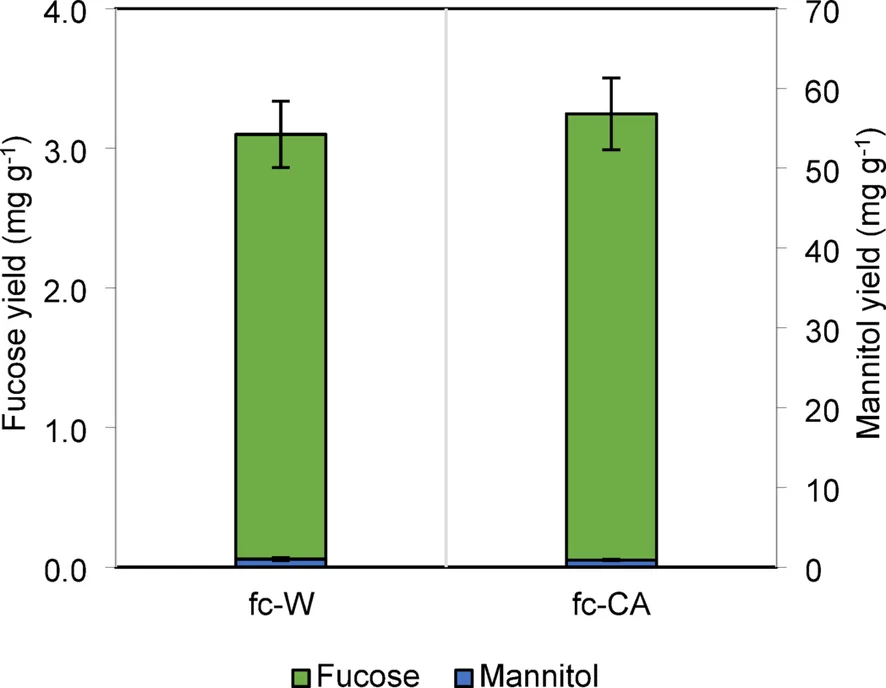
Fucose (fc) and mannitol (M) extraction yields from the aqueous (W) and citric acid pretreatments (CA)

As a first step in the sequential fractionation strategy, aqueous pretreatment was investigated to recover the water-soluble components fucoidan and mannitol prior to DES extraction. Mild acidic conditions have been reported to improve fucoidan extraction while promoting the conversion of alginate to alginic acid, potentially facilitating its recovery in subsequent fractionation steps (Saji et al. 2022, Fawzy and Gomaa 2021). Accordingly, water and citric acid-assisted pretreatments were compared to determine whether acidification could enhance the recovery of water-soluble compounds while influencing the subsequent DES-based extraction. A mild citric acid concentration of 50 mM was selected and applied at 35 °C for 1 h under light-protected conditions, thereby limiting exposure of the biomass to harsh processing conditions. Fucose, used as a proxy for fucoidan, and mannitol were quantified to evaluate the performance of both pretreatments (Fig. 2).

The extraction yields of both compounds were not significantly affected by the type of pretreatment. Mannitol yields were 58.5–59.3 mg g^− 1^, following water and citric acid pretreatment, respectively, whereas corresponding fucose yields were 3.1 and 3.2 mg g^− 1^. The comparable mannitol recovery under both conditions can be explained by its high water solubility and cellular localisation. As the low-molecular-weight polyol is predominantly located in the cytoplasm and, to a lesser extent, in vacuoles, mannitol can readily diffuse into the aqueous phase without requiring extensive biomass disruption (Groisillier et al. 2015). However, the resulting fucoidan-rich fraction was not exclusively composed of fucoidan, as mannitol was also detected at 1.0 ± 0.2 and 0.9 ± 0.1 mg g^− 1^ following water and citric acid pretreatment, respectively. This co-extraction reflects the limited selectivity of aqueous fractionation towards chemically distinct but water-soluble components (Graiff et al. 2016).

In contrast to mannitol, enhanced fucoidan recovery was anticipated under citric acid pretreatment (Fawzy and Gomaa 2021). However, no significant differences were observed between the two treatments. The absence of an acid-assisted improvement may be attributed to the deliberately mild conditions applied (50 mM), particularly the low temperature, which likely limited cell-wall disruption and fucoidan solubilisation. Higher citric acid concentrations (3%, approximately 156 mM) have indeed been reported to enhance the sequential extraction of fucoidan and alginate (Fawzy and Gomaa 2021). However, whether these more severe conditions affect the recovery or stability of fucoxanthin, the target compound in the subsequent extraction step, was not evaluated. Thus, increasing pretreatment severity solely to maximise fucoidan recovery may not necessarily benefit the overall sequential fractionation process.

The next step focused on the DES-based biphasic system designed for fucoxanthin recovery. Before experimentally evaluating this system, COSMO-RS was used to predict the affinity of fucoxanthin for the individual solvent phases and its resulting partitioning behaviour.

### Predicting fucoxanthin partition in the biphasic system

COSMO-RS calculations revealed a very low affinity of fucoxanthin for the BU phase, with a predicted capacity of 9.7 × 10^−6^ (Table 3). This affinity decreased further as the water content of the BU phase increased, with capacity values declining from 2.1 × 10^−8^ at 25% water to 3.3 × 10^−11^ at 65% water. This trend indicates that increasing the aqueous character of the BU phase progressively disfavours fucoxanthin solubilisation, in agreement with its predominantly hydrophobic molecular structure (Fig. S1).

In sharp contrast, fucoxanthin exhibited a high predicted affinity for the fatty-acid-based DES, with a capacity of 2.1 × 10^3^. The difference of several orders of magnitude between the FA and BU phases therefore provides a strong thermodynamic driving force for fucoxanthin partitioning towards the FA phase. This selectivity was further reflected by the predicted partition coefficient of the FA-BU45 system (K = 3.8 × 10^12^), supporting the suitability of this solvent combination for directing fucoxanthin into the fatty-acid-rich phase.

While COSMO-RS established the thermodynamic basis for selective fucoxanthin partitioning, practical application of this concept requires the two DES phases to coexist as a stable biphasic system. The phase-forming behaviour of the FA-BU mixtures was therefore investigated experimentally.

**Table 3:** Calculated capacities and partitioning of fucoxanthin in the different DES systems

	Capacity $\left( {{1 \mathord{\left/ {\vphantom {1 {\gamma _{i}^{\infty } }}} \right. \kern-\nulldelimiterspace} {\gamma _{i}^{\infty } }}} \right)$	$K_{{FA/BU}}$
BU	9.7 × 10^−6^	2.2 × 10^8^
BU25	2.1 × 10^−8^	1.0 × 10^11^
BU45	5.5 × 10^−10^	3.8 × 10^12^
BU65	3.2 × 10^−11^	6.5 × 10^13^
Dodec: Oct	2.1 × 10^3^	–

### FABU system behaviour

#### Feasibility of the biphasic system formation

**Fig. 3:**
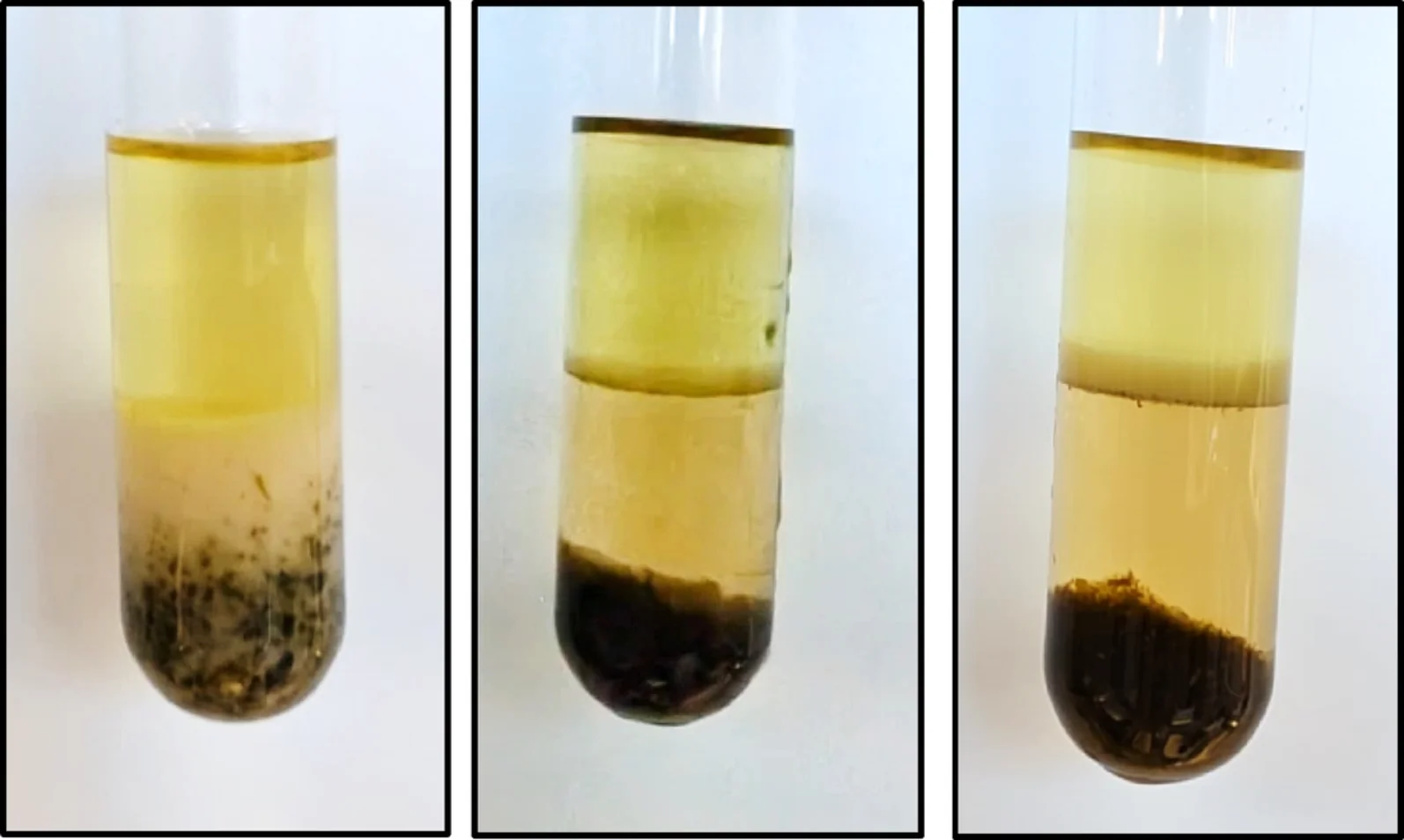
Biphasic systems at increasing water contents (from left to right): 25, 45, and 65% w/w

The ability of the FA and BU DES to form a biphasic system was evaluated across BU formulations containing different water contents. Following centrifugation, clear phase separation was observed for all formulations (Fig. 3), confirming that the two DES phases were mutually incompatible under the tested conditions. However, water content strongly influenced the physical state of the BU-rich phase. While the BU + 45 and BU + 65 systems remained fully liquid, partial solidification occurred in the hydrophilic phase of BU + 25. These results indicate that phase separation alone is insufficient to ensure an operational biphasic system and that a minimum water content is required to maintain the fluidity of the BU phase.

The requirement for a minimum water content can be attributed to the dual role of water as a structural disruptor and plasticiser of the betaine–urea hydrogen-bond network. Below a critical hydration level, stronger interactions between the DES constituents can favour structural ordering and ultimately crystallisation (Nava-Ocampo et al. 2021, Al Fuhaid et al. 2023). In the present biphasic system, this behaviour may be further influenced by interactions between the BU and hydrophobic FA phases, although the extent of such interactions was not investigated. In previous work, the BU DES was initially prepared with 15% (w/w) water, which increased to approximately 25% (w/w) upon contact with the seaweed due to its intrinsic moisture content (Reynaga-Navarro et al. 2026). Under these conditions, the resulting hydration level was sufficient to maintain the DES in a liquid state and prevent crystallisation.

The crystallisation observed at lower water contents upon contact with the FA phase suggests that phase interactions may alter the balance of intermolecular interactions required to maintain the BU DES in a liquid state. Fatty-acid carboxyl groups can interact through hydrogen bonding with urea and with functional groups of zwitterionic betaine, potentially competing with the interactions that stabilise the original betaine–urea network. Similar interactions have been reported in betaine-based DES systems used for palmitic acid removal from oils, supporting the affinity between fatty acids and betaine-based solvent components (Zahrina et al. 2018). At the same time, redistribution of water between the two phases may reduce the effective hydration of the BU-rich phase (Kivelä et al. 2022). Together, these competitive interactions and changes in water distribution could shift the intermolecular balance of the BU phase away from the composition required to maintain a stable liquid DES, thereby favouring crystallisation when the initial water content is insufficient.

Although the formation of two distinct liquid phases established the physical feasibility of the FABU system, phase formation alone does not guarantee efficient or selective fucoxanthin recovery. The next step was therefore to determine whether the strong preferential partitioning towards the FA phase predicted by COSMO-RS translated into experimental extraction performance. Fucoxanthin recovery and its distribution between the two phases were consequently evaluated.

#### FABU extraction performance

**Fig. 4:**
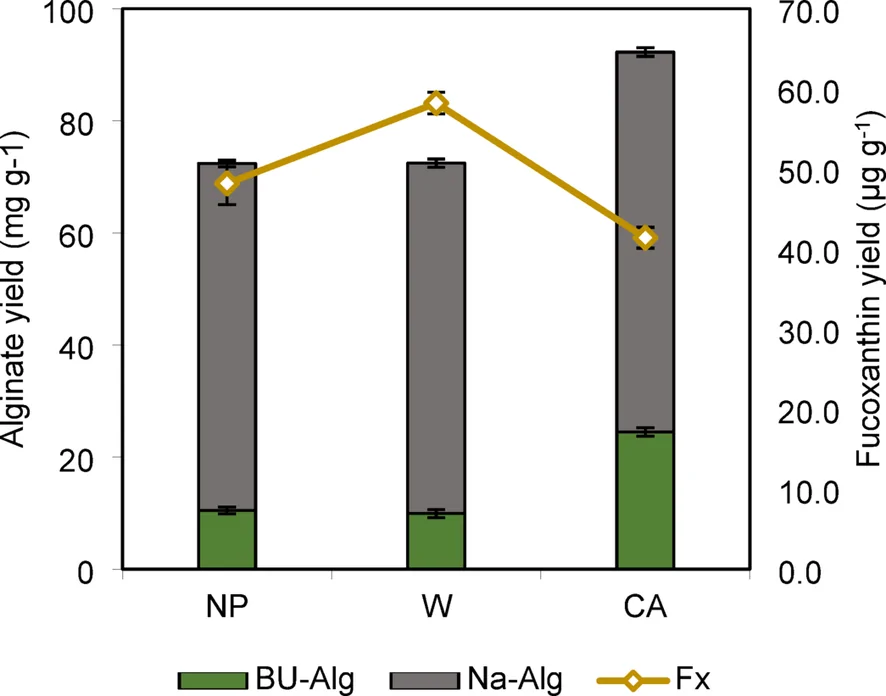
Extraction yields of BU-alginate (BU-Alg) (betaine-urea fraction), sodium alginate (Na-Alg) (alkaline extraction), and fucoxanthin (Fx) from the FABU system under different pretreatment conditions: no pretreatment (NP), aqueous (W), and citric acid (CA)

The extraction performance of the FABU system was evaluated using BU + 45, selected as a fully liquid biphasic formulation, and combined with the aqueous pretreatments described in Section “Aqueous seaweed pretreatment” To assess the distribution of the main target compounds across the process, alginate was recovered in two fractions: (i) directly from the hydrophilic BU-rich phase following biphasic extraction and (ii) by subsequent alkaline extraction of the residual biomass pellet (Sections “Biphasic extraction process” and “Pretreatments evaluation in combination with the biphasic system”). In parallel, fucoxanthin recovery in the hydrophobic FA-rich phase was quantified (Fig. 4).

The distribution of alginate between the two recovered fractions was consistent with previous observations for BU-based extraction (Reynaga-Navarro et al. 2026), with substantially less alginate recovered directly from the BU-rich phase than through subsequent alkaline extraction of the residual biomass. Interestingly, although citric acid pretreatment did not enhance fucoidan recovery under the mild conditions investigated (Section “Aqueous seaweed pretreatment”), it increased the yield of both BU-extracted alginate and the subsequently recovered sodium alginate (Na-Alg). This agrees with previous studies reporting improved alginate recovery following citric acid pretreatment (Fawzy and Gomaa 2021). The effect has been associated with the ability of citric acid to chelate divalent cations involved in cross-linking the alginate matrix, thereby disrupting ionic interactions within the cell wall and promoting conversion of alginate towards its protonated, alginic-acid form (Fawzy and Gomaa 2021). Such structural changes may increase the accessibility of alginate to the BU phase and facilitate its subsequent solubilisation under alkaline conditions, explaining the higher overall recovery observed after citric acid pretreatment.

To assess the chemical characteristics of the recovered alginate fractions, FTIR spectra were obtained for BU-Alg, Na-Alg, and the conventional alkaline extract. All fractions exhibited similar spectral profiles, with characteristic absorption bands around 1080 and 1020 cm^−1^ (Fig. S5), associated with vibrational modes of guluronic (G) and mannuronic (M) acid residues, respectively (Leal et al. 2008, Nesic et al. 2023), supporting the presence of alginate in the recovered fractions. The relative prominence of the mannuronic-associated band suggests a polymer rich in M residues, consistent with our previous characterisation of alginate extracted using the same betaine: urea DES (Reynaga-Navarro et al. 2026). However, FTIR analysis alone does not allow quantitative determination of the M/G ratio.

More extensive structural and functional characterisation was beyond the scope of the present study. Nevertheless, alginate recovered using BU DES has previously been characterised in terms of M/G ratio, average molecular weight, and rheological behaviour (Reynaga-Navarro et al. 2026, Hiemstra et al. 2025, Hiemstra et al. 2026), with the resulting polymers retaining high molecular weight and viscosity (Reynaga-Navarro et al. 2026). The introduction of the co-existing FA phase in the present FABU system could, however, influence alginate structure or functionality. Therefore, although the FTIR profiles indicate preservation of the main chemical features of alginate, further characterisation would be required to establish whether its molecular and rheological properties are fully retained in the biphasic process.

Fucoxanthin recovery showed relatively small variations among the three pretreatment conditions (no pretreatment, water pretreatment, and citric acid pretreatment), with yields ranging from 41.4 to 58.2 µg g^−1^ DW after a single extraction cycle (Fig. 4). Although the differences between treatments were statistically significant (*p* < 0.05), their magnitude was relatively small, indicating a limited practical influence of pretreatment on fucoxanthin recovery under the conditions investigated. The lower yield observed following citric acid pretreatment may be related to the sensitivity of fucoxanthin to acidic conditions, which can promote pigment degradation and isomerisation (Zhao et al. 2019, Yusof et al. 2025), thereby decreasing the amount available for subsequent extraction. Importantly, this moderate reduction in fucoxanthin recovery should be considered alongside the enhanced alginate recovery observed after citric acid pretreatment. Thus, selection of the pretreatment conditions involves a trade-off between the recovery of individual fractions and should ultimately be evaluated in the context of the overall multiproduct fractionation process.

Despite the strong thermodynamic preference of fucoxanthin for the FA phase predicted by COSMO-RS (Sect.  3.2), this affinity did not translate into high experimental extraction yields. This discrepancy suggests that fucoxanthin recovery was not primarily limited by its partitioning between the two solvent phases, but rather by its release from the seaweed matrix and subsequent mass transfer into the FA phase. Thus, kinetic and matrix-related limitations appear to dominate the overall extraction performance under the conditions investigated.

This interpretation is consistent with the wide variation in fucoxanthin recoveries reported for *S. latissima*, which depend strongly on biomass origin, harvesting season, pretreatment, solvent system, and extraction conditions (Cunningham et al. 2023, Marinho et al. 2019). For *S. latissima* harvested in May, fucoxanthin contents of approximately 300–600 µg g^− 1^ DW have been reported (Cunningham et al. 2023, Marinho et al. 2019), although these values were generally determined using methanol-based extraction and therefore represent a different extraction environment. Importantly, the limited recovery observed here cannot be attributed solely to an intrinsically lower extraction capacity of DES, as Kholany et al. (Kholany et al. 2025) demonstrated that a menthol-levulinic acid DES could recover more fucoxanthin from *S. latissima* than methanol. In the present FABU system, however, only approximately 12% of the fucoxanthin recovered by the methanol reference extraction was obtained. This comparatively low recovery supports the hypothesis that solvent affinity was not the principal limitation; rather, a substantial fraction of fucoxanthin likely remained associated with cellular and thylakoid membrane structures, restricting its release and subsequent transfer into the FA phase.

One factor that may contribute to these mass-transfer limitations is the high viscosity of the DES phases. Betaine-based DES are generally characterised by high viscosities, particularly at relatively low temperatures (Allahyari et al. 2025). Although increasing water content can substantially reduce the viscosity of the BU phase, transport within the biphasic system may additionally be influenced by interfacial interactions between the two DES phases and by the identity and rheological properties of the continuous phase (Pal 2001, DeRoussel et al. 2001). Such effects can increase resistance to mass transfer across the phase boundary and thereby slow the transport of fucoxanthin released from the biomass towards the FA-rich phase. Moreover, redistribution of water between the phases may modify their local solvent environments, potentially affecting both solvation and molecular transport (Kivelä et al. 2022).

This limitation may be particularly relevant for the FABU configuration compared with previously reported fatty-acid DES–salt systems, in which the second phase was predominantly aqueous (Hiemstra et al. 2025). Replacing this aqueous phase with a second DES creates a solvent-rich biphasic environment that is expected to exhibit greater resistance to molecular transport. Consequently, although the FABU configuration offers the advantage of combining two functional DES phases, this may come at the expense of slower transfer of compounds from the biomass to their thermodynamically preferred phase (Hu et al. 2023). The persistence of extractable compounds in the residual biomass, as revealed by the subsequent alkaline extraction step, provides further experimental support for incomplete mass transfer during the initial FABU extraction.

#### Fucoxanthin retainment in the biomass

Further evidence of incomplete fucoxanthin recovery emerged during the subsequent alkaline extraction of the residual biomass. Unexpectedly, a hazy, yellow-coloured extract was obtained (Fig. S2) suggesting that pigment-containing material remained in the biomass after FABU extraction and was released during alkaline treatment. This observation is consistent with the proposed limitation in fucoxanthin accessibility and mass transfer discussed above. *S. latissima* has been reported to contain fucoxanthin concentrations as high as 1.96 mg g^−1^ DW (Martins et al. 2021), highlighting the potentially substantial pigment pool present in the biomass. However, confirmation that the yellow colour observed after alkaline extraction originated specifically from residual fucoxanthin required further analysis.

**Fig. 5:**
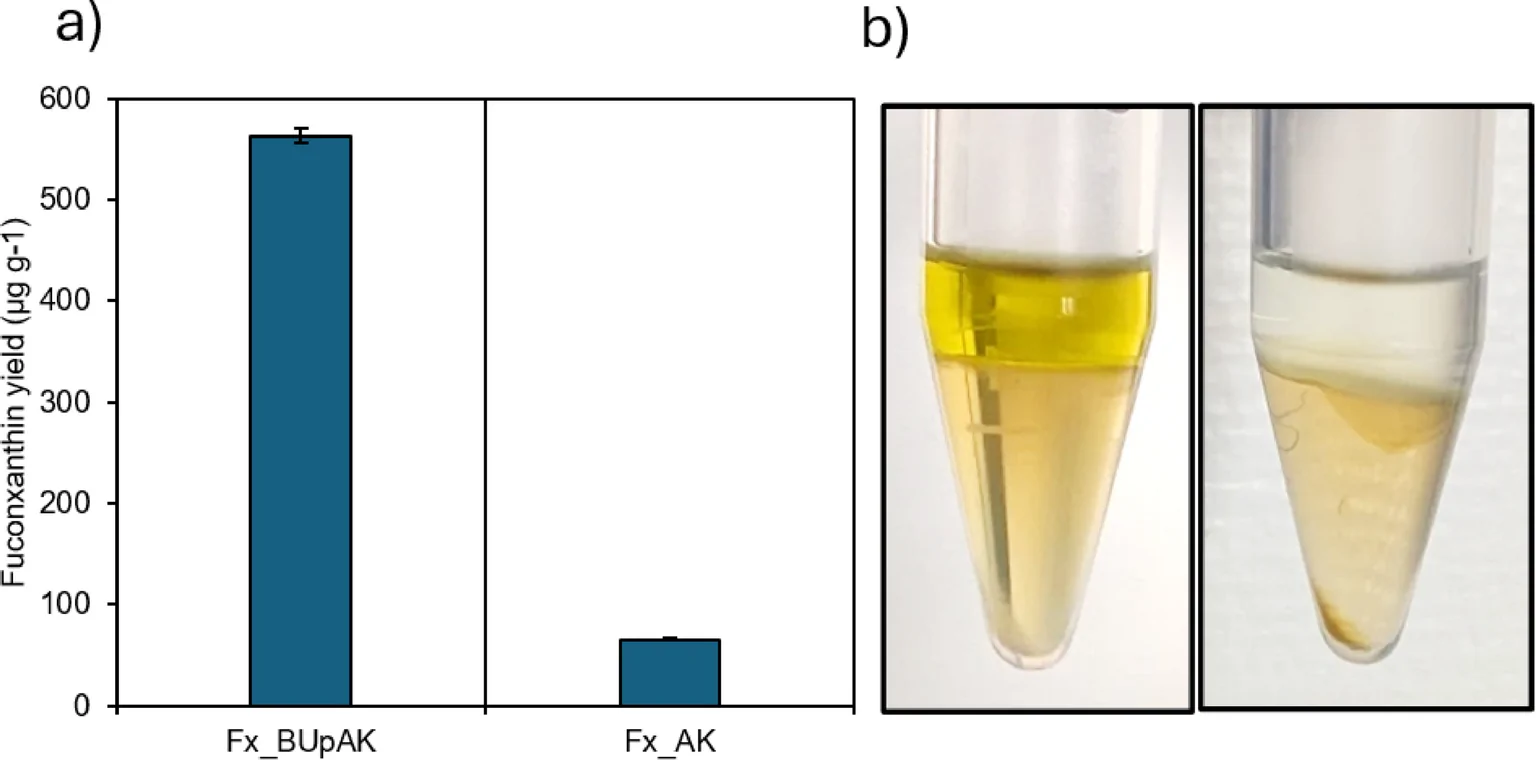
**a** Fucoxanthin extraction yields (Fx) in the alkaline extract after the FABU biphasic system extraction (BUpAK) and in the AK extract. **b** Fucoxanthin recovered in ethyl acetate (Top phases) from the alkaline extract from the FABU system (left) and from the conventional extraction process (right)

To verify whether the pigment retained in the biomass was fucoxanthin, the alkaline extract was subjected to liquid–liquid separation followed by fucoxanthin quantification. Remarkably, alkaline extraction of the residual biomass following FABU treatment produced an intensely yellow pigment-rich phase (Fig. 5b) and a fucoxanthin yield of 563.3 ± 7.7 µg g^−1^ DW (Fig. 5a). This recovery was substantially higher than that achieved directly in the FA phase during FABU extraction (Fig. 4). In contrast, direct sodium carbonate extraction of untreated biomass, used as a control, yielded only a pale ethyl acetate phase (Fig. 5b) and negligible amounts of fucoxanthin (Fig. 5a). The marked difference between untreated and FABU-treated biomass indicates that exposure to the biphasic DES system substantially enhanced the subsequent accessibility and release of fucoxanthin during alkaline extraction. Although the underlying mechanism was not directly investigated, the results are consistent with DES-induced alterations of the biomass matrix that facilitate pigment release.

During FABU extraction, the hydrophilic BU-rich phase may promote swelling and partial disruption of the seaweed matrix, thereby increasing pigment accessibility without itself favouring fucoxanthin solubilisation (Loow et al. 2017, Li et al. 2023). However, this matrix-modifying effect was apparently insufficient to enable extensive transfer of fucoxanthin into the FA-rich phase during the extraction period. Despite the strong affinity of fucoxanthin for the FA DES predicted by COSMO-RS (Table 3) and the efficient recovery previously reported for fatty-acid-based DES systems by (Hiemstra et al. 2025), a substantial fraction of the pigment remained associated with the biomass. Given the localisation of fucoxanthin within pigment–protein complexes of the thylakoid membranes (Hiemstra et al. 2025, Vothknecht and Westhoff 2001, Wang et al. 2019), its release likely requires disruption of cellular and membrane structures before favourable solvent–solute interactions can effectively drive extraction.

This discrepancy between predicted affinity and experimental recovery highlights an important limitation of thermodynamic screening when applied directly to complex biomass extraction. COSMO-RS describes the thermodynamic affinity of an accessible solute for a given solvent environment but does not account for the structural barriers, release kinetics, and mass-transfer limitations governing its liberation from the macroalgal matrix. Thus, high predicted solvent affinity does not necessarily translate into high extraction yield when accessibility of the target compound is the rate-limiting step.

This interpretation is further supported by previous work from our group showing that extraction kinetics in macroalgae are strongly governed by tissue density and biomass architecture (Agusman et al. 2026). In dense macroalgal tissues, enhanced cell permeabilisation alone was insufficient to ensure efficient compound recovery; extraction remained dependent on the rate at which the solvent penetrated the biomass and accessed the target structures (Agusman et al. 2026). Applied to the present system, these findings suggest that the limited direct recovery of fucoxanthin in the FA phase may arise not from insufficient solvent affinity, but from the restricted penetration and transport of the solvent through the seaweed matrix within the extraction time applied. A possible mechanism underlying this behaviour arises from the contrasting interactions of the two DES phases with the seaweed matrix. During FABU extraction, the hydrophilic betaine: urea (BU) phase may penetrate and hydrate the carbohydrate-rich matrix, promoting substantial biomass swelling (Reynaga-Navarro et al. 2026). Such hydration could create a locally water-rich environment that is readily accessible to the BU phase but less favourable for penetration of the highly hydrophobic FA phase. Consequently, although COSMO-RS predicts very high partition coefficients (10^11^–10^13^) towards the FA phase, physical separation between the pigment-containing structures and the FA solvent could restrict the molecular contact required for fucoxanthin transfer (Azmir et al. 2013). Fucoxanthin may therefore remain associated with internal thylakoid structures despite its strong thermodynamic affinity for the FA phase.

The subsequent sodium carbonate treatment appears to overcome this accessibility barrier, as evidenced by the substantial fucoxanthin recovery observed only after alkaline treatment of FABU-extracted biomass. Alkaline treatment may further disrupt the hydrated biomass and membrane-associated structures, facilitating release of the retained pigment and its subsequent recovery during liquid–liquid separation. Although direct structural evidence would be required to confirm this mechanism, the experimental observations support a sequential effect in which the FABU treatment increases biomass accessibility, while the subsequent alkaline step enables release of otherwise inaccessible fucoxanthin.

This matrix-controlled behaviour also helps explain the markedly different extraction profiles of the seaweed components investigated in this study. Whereas mannitol and fucoidan-derived fucose were readily recovered during mild aqueous pretreatment (Sect.  3.1), efficient fucoxanthin release required more extensive modification of the biomass matrix. This contrast can be related to the distinct spatial distribution and compartmentalisation of these compounds within the macroalgal tissue. Mannitol is a low-molecular-weight, water-soluble polyol predominantly located in the cytoplasm and vacuoles (Hosseini et al. 2024), whereas fucoidan is a water-soluble sulphated polysaccharide associated with the extracellular cell-wall matrix (Li et al. 2008). Their relatively high aqueous accessibility therefore enables substantial recovery under mild water or citric acid treatment without extensive disruption of internal cellular structures.

Fucoxanthin, in contrast, is hydrophobic and localised within chloroplast thylakoid membranes, where it is associated with lipid–protein complexes and physically protected by the surrounding cellular and cell-wall structures. Its extraction therefore requires not only a solvent with favourable thermodynamic affinity but also sufficient access to these internal pigment-containing structures. The present results suggest a cooperative but sequential role of the FABU system: the hydrophilic BU phase may hydrate and swell the carbohydrate-rich matrix, while penetration of the hydrophobic FA phase into this hydrated environment remains restricted. Subsequent alkaline treatment may then further disrupt the preconditioned biomass, enabling release of the retained fucoxanthin. Thus, rather than acting solely as a direct extraction medium, FABU may also function as a biomass-conditioning step that facilitates subsequent pigment recovery.

Given the poor water solubility of fucoxanthin (Ma et al. 2019, Fernandes and Mamatha 2023) and its limited solubility in aqueous sodium carbonate (Fig. S4), the high pigment recovery observed in the alkaline extract is unlikely to arise solely from molecular solubilisation in the aqueous phase. Instead, the co-recovery of fucoxanthin with alginate may involve a colloidal or emulsification-mediated mechanism. Residual fatty acids retained in the biomass after FABU treatment could participate, together with membrane-derived proteins and phospholipids, in the formation of dispersed structures capable of incorporating and transporting hydrophobic fucoxanthin into the aqueous extract (Hiemstra et al. 2025, Zhao et al. 2019). This mechanism could be further promoted under alkaline conditions, where fatty acids are partially converted into their corresponding sodium salts, increasing their surfactant character and favouring emulsification (Niraula et al. 2004, Hasenhuettl and Hartel 2019). Such dispersed systems may additionally contribute to pigment stabilisation, although the extent of this protective effect depends strongly on emulsion composition and stability (Ma et al. 2019).

The elevated temperature applied during alkaline extraction may have further facilitated pigment release from the preconditioned biomass, while previous studies indicate that fucoxanthin can retain relatively good stability under alkaline conditions (Fernandes and Mamatha 2023, Yusof et al. 2022). Taken together, these observations suggest that FABU treatment primarily enhances the accessibility of initially entrapped fucoxanthin, whereas the subsequent alkaline step promotes its release and apparent aqueous recovery, potentially through a combination of matrix disruption and emulsification-mediated transport. This interpretation is consistent with the negligible fucoxanthin recovery obtained when untreated biomass was subjected directly to the same alkaline extraction.

A potential limitation of this emulsification-mediated recovery mechanism is the formation of stable dispersions that may hinder subsequent phase separation and product purification. An additional solvent-based separation step may therefore be required to recover fucoxanthin while simultaneously facilitating alginate purification. Ethanol represents a promising option because it can precipitate alginate while maintaining fucoxanthin in the solvent phase, thereby enabling separation of the two products, followed by ethanol recovery and pigment concentration through solvent evaporation (Bojorges et al. 2023). Alternatively, ethyl acetate, as evaluated at laboratory scale in the present study, could be used to selectively transfer fucoxanthin from the aqueous alkaline fraction into an organic phase. Ethyl acetate is considered among the more environmentally favourable conventional organic solvents, and its relatively high volatility facilitates subsequent solvent recovery (Vergaelen et al. 2020). The choice between these strategies would ultimately depend on separation efficiency, solvent recyclability, and their integration within the overall fractionation process.

Alternatively, emulsion destabilisation could be pursued without introducing an additional organic solvent. One possible strategy is a controlled decrease in pH (Xiao et al. 2025), which would re-protonate fatty-acid salts, reduce their surfactant character, and thereby promote emulsion destabilisation. A gradual pH decrease could potentially be achieved through CO₂ sparging (Ma et al. 2021), offering a mild and readily controllable acidification route. However, the applicable pH window would need to be carefully defined, as excessive acidification could simultaneously induce alginate precipitation in the form of alginic acid (Bojorges et al. 2023) and compromise fucoxanthin stability (Yusof et al. 2022). Alternatively, increasing the ionic strength, for example through NaCl addition, could facilitate phase separation by screening electrostatic interactions between dispersed droplets, thereby promoting their aggregation and eventual coalescence (Xiao et al. 2025). Both strategies therefore offer potential solvent-free routes for resolving the pigment-containing dispersion, but their compatibility with simultaneous alginate recovery and fucoxanthin preservation would require further optimisation.

Depending on the targeted fucoxanthin purity and final application, additional purification steps, potentially including chromatographic techniques, may be required (Pajot et al. 2023). Importantly, the suitability of these downstream strategies should not be assessed solely in terms of fucoxanthin recovery and purity. Their impact on the co-recovered alginate must also be considered, particularly with respect to polymer yield, molecular structure, and functional properties. An effective separation strategy should therefore balance the recovery and quality of both product fractions while remaining compatible with solvent recycling and overall process integration.

**Fig. 6:**
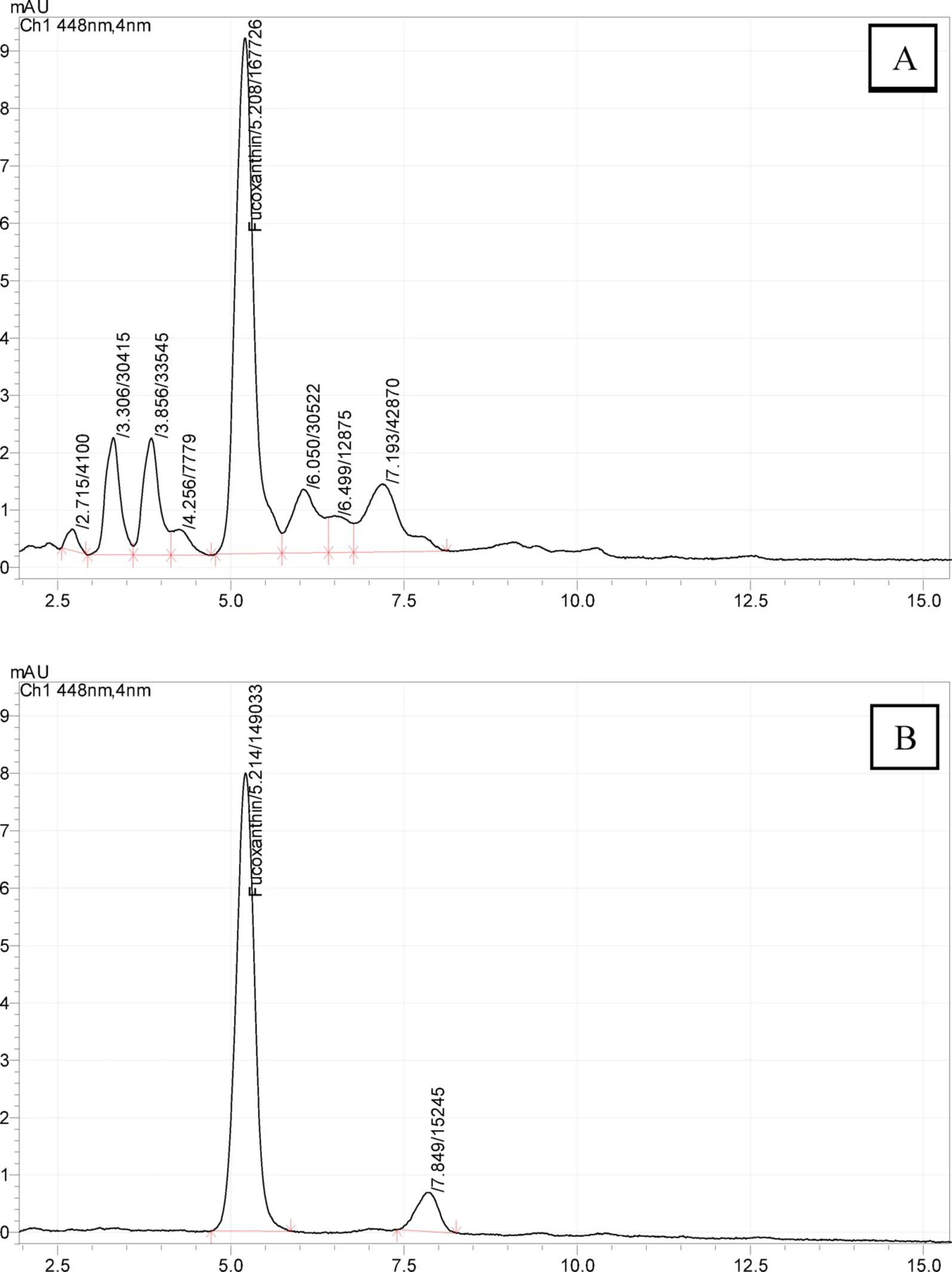
**A** Fucoxanthin recovered with methanol from the pellet residue after the conventional alkaline extraction. **B** Fucoxanthin recovered with ethyl acetate from the alkaline extraction after FABU treatment. More details regarding the UV-vis spectra of secondary peaks can be found in the Supplementary Material Section

For comparison, the residual biomass obtained after conventional acid–alkaline alginate extraction was subsequently subjected to methanol extraction to determine the amount and integrity of the remaining fucoxanthin. A yield of 328.9 ± 2.2 µg g^− 1^ DW was recovered, demonstrating that a substantial fraction of the pigment remained in the biomass even after conventional alginate extraction. However, chromatographic analysis revealed marked differences in pigment composition. In addition to the main peak assigned to all-trans-fucoxanthin (RT ≈ 5.2 min), several secondary peaks with similar absorption profiles eluted both before and after the main peak. These peaks, tentatively attributed to fucoxanthin isomers and/or degradation products (Agung Wibowo et al. 2022), accounted for approximately 39.5 ± 0.5% of the total chromatographic area (Fig. 6A), indicating substantial modification of the pigment during conventional acid–alkaline processing. In contrast, only minor secondary peaks were observed for fucoxanthin recovered from the alkaline fraction following FABU treatment (Fig. 6B).

Further evidence of pigment alteration in the conventional process was provided by the absorption spectra of the secondary chromatographic peaks, which exhibited a hypsochromic shift of the main absorption band towards approximately 420–430 nm (Fig. S3) (Llansola-Portoles et al. 2017). Such spectral changes are consistent with alterations of the conjugated chromophore associated with fucoxanthin isomerisation and/or degradation (Llansola-Portoles et al. 2017). Together, these results suggest that although both processing routes leave a fraction of fucoxanthin associated with the residual biomass, FABU treatment provides a milder chemical environment that enhances subsequent pigment accessibility while better preserving its structural integrity.

The ability of DES to enhance carotenoid stability has been reported for several pigment–solvent systems. Xu et al. (Xu et al. 2022) demonstrated that fucoxanthin extracted from the microalga *Tisochrysis lutea* using a thymol: dodecanoic acid DES remained stable for 11 days without significant degradation. Similarly, lutein extracted from *Scenedesmus* sp. using NADES showed greater stability than in methanol under elevated temperature and light exposure, an effect attributed to hydrogen-bonding and van der Waals interactions between the solvent components and pigment molecules (Fan et al. 2022). A comparable protective effect was reported by Yang et al. 2023 for canthaxanthin extracted from *Chromochloris zofingiensis* using an octanoic acid: decanoic acid DES, where reduced pigment aggregation and a more homogeneous solute distribution were observed relative to conventional solvents. Collectively, these studies indicate that the DES environment can influence not only carotenoid solubilisation but also pigment stability after extraction. Given the fatty-acid-based composition of the hydrophobic phase used here, similar solvent–pigment interactions may have contributed to the comparatively limited alteration of fucoxanthin observed following FABU treatment.

Taken together, the present results reveal a potential dual role of the FABU system within the sequential fractionation process. Although the strong thermodynamic preference predicted for fucoxanthin towards the FA phase did not translate into high direct extraction yields, FABU treatment substantially increased the accessibility of the pigment for subsequent recovery under alkaline conditions. At the same time, the chromatographic profiles showed considerably fewer potential isomerisation/degradation products than after conventional acid–alkaline processing, indicating better preservation of fucoxanthin integrity. Rather than functioning solely as a direct biphasic extraction medium, FABU may therefore combine biomass conditioning with a protective solvent environment, effectively decoupling matrix disruption from final pigment recovery. This dual functionality offers a different route to process intensification, in which solvent selection is used not only to drive thermodynamic partitioning but also to control biomass accessibility and product stability across sequential extraction steps.

### Mass balance of the proposed design

To evaluate the overall performance of the integrated fractionation process, the recovery of each target compound was calculated relative to its initial content in the biomass (Table S1). Alginate recovery was lower than that previously reported by Reynaga-Navarro et al. (Reynaga-Navarro et al. 2026) using BU DES. This difference may partly arise from the lower extraction temperature applied in the present study (35 °C compared with 55 °C). Besides slowing molecular diffusion, lower temperatures increase DES viscosity, thereby imposing additional mass-transfer limitations. Increasing temperature or otherwise improving mass transfer could therefore provide opportunities to enhance alginate recovery, although such optimisation would need to preserve the quality of the co-recovered compounds. A similar opportunity for optimisation was identified for fucoidan, for which fucose-based recovery reached only 32%. This relatively low recovery is consistent with the mild pretreatment conditions selected here, particularly the low citric acid concentration compared with concentrations previously reported as optimal for fucoidan extraction (Fawzy and Gomaa 2021). Mannitol, in contrast, reached approximately 80% recovery, reflecting its high water solubility and accessibility under mild aqueous conditions (Groisillier et al. 2015). Taken together, these recoveries illustrate an inherent trade-off in the integrated process: the mild conditions selected to limit degradation of sensitive compounds, particularly fucoxanthin, did not maximise the extraction yield of every individual fraction. The current process should therefore be regarded as a compromise between multiproduct recovery and preservation of product integrity rather than as an individually optimised extraction of each component. Notably, the cumulative fucoxanthin recovered across the integrated process exceeded the amount quantified using the conventional methanolic reference extraction, resulting in an apparent relative recovery above 100%. This value should not be interpreted as a true mass recovery exceeding the initial fucoxanthin content of the biomass; rather, it indicates that the methanolic reference extraction was not exhaustive under the conditions employed. Importantly, the result demonstrates that sequential processing enabled access to a fucoxanthin fraction that remained unrecovered by the methanol benchmark, further supporting the role of matrix accessibility in determining pigment recovery from *S. latissima*.

### FABU as a powerful pretreatment

#### Proposed permeabilization mechanism

For structurally complex biomass such as seaweed, favourable solute partitioning alone is insufficient to ensure efficient extraction. The solvent must first access and modify the cellular matrix sufficiently to expose the target compounds to the phase for which they have a thermodynamic affinity. The results obtained with FABU illustrate this interplay between solvent–solute affinity and biomass accessibility.

Confocal microscopy provides direct evidence that FABU treatment altered the architecture of the seaweed tissue (Table S2; Fig. S6). Hydrated control samples exhibited a larger relative cell area compared with the intercellular cell-wall space, whereas FABU-treated samples showed a reduction in the outer cell-wall layer. This change is consistent with partial removal of cell-wall material, including alginate, and suggests increased accessibility of the internal tissue. Such structural modification provides a plausible physical basis for the enhanced release of intracellular fucoxanthin observed during subsequent alkaline extraction.

This interpretation is supported by the known spatial heterogeneity of alginate within brown-algal cell walls. Jones et al. 2025 reported that mannuronate-rich alginate epitopes (antibody-recognized structural regions) occur predominantly at the outer cell-wall surface and in intercellular regions, whereas guluronate-rich epitopes extend across the structural cell wall. Similarly, in *S. latissima* embryos, (Boscq et al. 2025) observed spatially distinct distributions of mannuronate- and guluronate-rich alginate domains during cell-wall development. Together, these studies demonstrate that alginate is not uniformly distributed throughout brown-algal tissues, providing a structural basis for preferential mobilisation of specific alginate fractions during sequential extraction. Although the confocal microscopy performed here clearly indicates changes in tissue architecture following FABU treatment, complementary techniques such as scanning electron microscopy (SEM) would be required to directly resolve changes in cell-wall surface morphology and further substantiate the proposed permeabilisation mechanism.

Further support for selective cell-wall fractionation comes from our previous sequential extraction study using betaine: urea DES. The first alginate fraction recovered directly with betaine: urea (BU-1) represented only part of the total alginate and exhibited an M/G ratio of approximately 2.2, indicating preferential recovery of a mannuronate-rich fraction. Subsequent water and alkaline fractions showed progressively lower M/G ratios of approximately 1.5 and 1.2, respectively. Most alginate was recovered only during the final alkaline step, while the complete sequential BU process achieved an overall recovery of 75.1 ± 2.2%, compared with 63.5 ± 1.6% for the conventional acid–alkaline process. These observations indicate that betaine: urea does not uniformly solubilise the structural alginate matrix. Rather, it preferentially mobilises a more accessible, mannuronate-rich fraction while modifying the remaining matrix in a manner that facilitates subsequent alginate recovery.

Integrating the microscopy observations with the sequential extraction and M/G fractionation data therefore supports a mechanism in which FABU progressively modifies, rather than completely disrupts, the seaweed cell-wall matrix. The BU-rich phase hydrates and swells the carbohydrate-rich tissue, while preferential mobilisation of accessible alginate domains partially loosens the outer matrix and increases access to internal cellular structures. More tightly integrated alginate remains associated with the biomass and is subsequently solubilised under alkaline conditions. Importantly, this progressive matrix modification also provides a mechanistic explanation for the fucoxanthin results: the pigment remains largely inaccessible to the FA phase during FABU extraction but becomes substantially more accessible once the preconditioned biomass undergoes alkaline treatment. Thus, the principal contribution of FABU may lie not only in direct compound partitioning, but in creating a more accessible biomass architecture that enables sequential recovery of chemically and spatially distinct product fractions.

#### Process perspective

Translating the proposed integrated extraction scheme into a circular seaweed biorefinery will require efficient recovery and reuse of both DES phases. Although solvent recycling was not investigated in the present study, previous work from our group has demonstrated the multi-cycle recovery of the individual solvent systems in related extraction configurations. Betaine: urea (BU) DES has been recovered and reused for at least five consecutive cycles while simultaneously enabling alginate separation, using both temperature-responsive aqueous two-phase systems (ATPS) (Hiemstra et al. 2025) and DES–salt ATPS with direct interfacial alginate precipitation (Hiemstra et al. 2026). Similarly, the fatty-acid (FA) DES has been reused for up to seven extraction cycles in a circular three-phase partitioning (TPP) configuration coupled with pigment recovery (Hiemstra et al. 2025). Together, these studies demonstrate that both solvent components can, independently, be integrated into recyclable extraction schemes and therefore provide a technical basis for developing solvent recovery within an integrated FABU process. However, simultaneous recovery and reuse of both phases from the FABU system remains to be demonstrated.

Beyond technical feasibility, the viability of an integrated seaweed biorefinery must ultimately be assessed at process scale, accounting for solvent recovery, product purification, energy requirements, and the interactions between sequential unit operations (Herrera Barragán et al. 2022, Azzouz and Hayyan 2024). DES-based processes may introduce specific engineering challenges associated with their physicochemical properties, particularly viscosity, which can affect mixing, pumping, mass transfer, and equipment design and thereby influence both capital and operating costs (Hansen et al. 2021, Rente et al. 2022). The costs associated with DES preparation, make-up, and losses during recycling must likewise be quantified, as even highly recyclable solvents require sufficiently high recovery rates to remain economically competitive (Wang et al. 2025, Lobato-Rodríguez et al. 2023).

Importantly, process feasibility cannot be evaluated independently of the properties and intended applications of the recovered products. The economic value of alginate, fucoxanthin, fucoidan, and other fractions depends not only on recovery yield, but also on purity, structural integrity, functionality, and the market targeted (Azzouz and Hayyan 2024). Higher processing costs may therefore be justified if the integrated process preserves or enhances product functionality while simultaneously enabling recovery of multiple value streams from the same biomass. In this context, the present study provides a technical proof of concept for such an integrated approach, while techno-economic assessment will be required to determine whether the benefits of multiproduct recovery and product preservation compensate for the additional process complexity.

Although further optimisation of the FABU system is required, the results establish a promising basis for integrated multiproduct seaweed fractionation in which biomass conditioning, solute accessibility, and thermodynamic partitioning are treated as complementary rather than simultaneous requirements. By strategically decoupling matrix disruption from final product recovery, the proposed approach provides a flexible framework for sequentially accessing compounds with markedly different physicochemical properties and cellular localisations.

## Conclusion and recommendations

A DES-based biphasic system (FABU) was evaluated for the integrated co-extraction of alginate and fucoxanthin from *Saccharina latissima*. This system combined a hydrophobic phase (dodecanoic acid-octanoic acid, FA) targeted for fucoxanthin extraction with a hydrophilic phase (betaine-urea-water, BU) for alginate recovery.

Unexpectedly, fucoxanthin partitioning into the FA phase was limited (41.4–58.2 µg g^− 1^), contradicting COSMO-RS predictions regarding both thermodynamic affinity (capacity of 2.1 × 10^3^ for FA) and the partition coefficient (K_FA/BU_ 3.8 × 10^12^). Remarkably, a high fucoxanthin concentration (563.3 ± 7.7 µg g^− 1^) was recovered during the subsequent alkaline treatment, which was originally designated for complete alginate recovery. This indicates that the FABU system primarily functions as a cell permeabilisation medium rather than an active liquid-liquid equilibrium partitioning platform.

This discrepancy between theoretical thermodynamic affinity and experimental extraction behaviour highlights that partitioning data alone is insufficient to describe extraction performance in complex biomass matrices. Instead, factors such as biomass accessibility, mass transfer limitations, and system viscosity play a dominant role. These findings demonstrate that disruption and solubilisation can be effectively decoupled, with the FABU system enhancing accessibility while the subsequent alkaline step enables compound recovery.

Chromatographic profiles further suggested improved fucoxanthin preservation compared to conventional acid-alkaline extraction, indicating the DES was likely acting as a preservative medium.

Overall, these results reveal a clear decoupling between cell permeabilization capacity and solubilisation in DES-based systems. Rather than a limitation, this uncovers the potential of DES biphasic systems as powerful pretreatment platforms for integrated seaweed biorefineries.

Future work should address solvent recyclability and assess process feasibility through techno-economic and life cycle analyses and complete the characterisation of the extracted compounds.

## Supplementary Information

Below is the link to the electronic supplementary material.

Supplementary Material 1.

## Data Availability

Data will be made available on reasonable request.
