# Supplementary material for: Disruption or partitioning? Deep eutectic solvent biphasic systems for integrated seaweed biorefineries
Source: Bioresour Bioprocess. 2026 Sep 25;13(1):141. doi: 10.1186/s40643-026-01139-9 (PMC13615259; doi:10.1186/s40643-026-01139-9)
Supplement: Supplementary file 1 [file 40643_2026_1139_MOESM1_ESM.docx]

SUPPLEMENTARY MATERIAL

Figure S1. Molecular structure of fucoxanthin. Blue regions represent the hydrogen bond donating areas, red regions represent the hydrogen bond accepting areas, and green regions represent the non-polar surface areas.

Figure S2. Alkaline extract from the FABU system (left) and from the conventional process (right). Extract from the FABU system was browner than the extract from the conventional method, indicating the presence of a higher concentration of fucoxanthin.

Fx

C

D

B

A

Figure S3. UV-Vis absorption spectra of fucoxanthin (Fx) and the four secondary peaks (A–D) observed in the chromatogram shown in Figure 7A. Peaks A, C, and D exhibited a hypsochromic shift of the main absorption band to approximately 420-430 nm, suggesting degradation of the conjugated polyene chain [67]. In contrast, peak B showed a maximum absorption at 453 nm, consistent with the presence of a potential fucoxanthin isomer [66].

|  | Capacity (1/γ) |
| --- | --- |
| BU (1:2) | 9.65065e-06 |
| BU25 (1:2) | 2.09486e-08 |
| BU45 (1:2) | 5.51086e-10 |
| BU65 (1:2) | 3.24802e-11 |
| Dodec:Oct (1:1) | 2100 |
| Na2CO3 (0.1 M) | 1.47399e-12 |
| Na2CO3 (4 M) | 47846.9 |

Figure S4. Calculated capacities of DES and sodium carbonate for fucoxanthin. From left to right: BU, betaine-urea DES. The numbers on the right indicate the water content (% w/w), while the values in parentheses represent the molar ratios. Dode:Oct, docecanoic acid-octanoic acid DES; Na_2_CO_3_ corresponds to sodium carbonate solutions at 0.1 and 4 M.

**FTIR analysis**

To evaluate the alginate recovered from the DES extract, acid alkaline extract and the alkaline extract after DES treatment, FTIR (Fourier-transform infrared) profiles were recorded over a spectral range of 400–4000 cm⁻¹ using a Nicolet Summit X ATR-FTIR spectrometer (Thermo Fisher Scientific). Prior to analysis, polymers were isolated from the extracts by precipitation with a ethanol 80% (v/v) with 1% (w/v) sodium chloride, followed by two sequential washing steps using 80% (v/v) ethanol [11]. The gathered precipitates were subsequently dried under a continuous nitrogen stream before being subjected to FTIR characterisation.

Figure S5. FTIR spectra of alginate extracted with acid-alkaline conventional extraction (red), alkaline extraction after BU treatment (blue) and DES BU (grey) using the FABU biphasic system.

Table S1. Mass balance of extracted compounds in the different fractions along the proposed FABU process.

| **Fraction** | **Alginate  (mg g-1)** | **Fucose  (mg g-1)** | **Mannitol  (mg g-1)** | **Fucoxanthin  (µg g-1)** |
| --- | --- | --- | --- | --- |
| Seaweed | 100% | 100% | 100% | 100% |
| Pretreatment | n.d. | 32% | 80% | 0% |
| FABU | 8% | n.d. | n.d. | 12% |
| AK-extraction | 23% | n.d. | n.d. | 167% |

**Microscopy of treated biomass**

Samples were imaged with a Leica Stellaris 5 confocal microscope (Leica) using a 20× objective (**Figure S6**). Image acquisition was performed with a white light laser (WLL) at excitation wavelengths of 485 and 680 nm, with emission bands set at 675–695 and 710–740 nm, respectively. A transmitted-light detector was used to acquire a brightfield channel simultaneously. Images were acquired at a resolution of 1024 × 1024 pixels with a scan speed of 100 Hz. Image processing and analysis were performed using LAS X software (Leica).

D

C

B

A

Figure S6. Confocal microscopy images of treated seaweed. Untreated (A); acid-alkaline treated (B); FABU treated (C); FABU-alkaline (D).

Table S2 Relative area of seaweed cells compared to cell walls.

| Sample | Area fraction  (Intra/inter) %^a^ |
| --- | --- |
| Water | 66 ± 6 |
| FABU | 54 ± 1 |

^a^ Values represent the mean of three independent images analysed using ImageJ. The percentages for the conventional acid-alkaline and FABU-alkaline samples could not be determined due to insufficient image contrast.
